# Clinical and pathological features of hepatoid carcinoma of the ovary

**DOI:** 10.1186/1477-7819-11-29

**Published:** 2013-01-30

**Authors:** Lina Wang, Yanping Zhong, Lingling Sun, Heng Zhou, Wei Chen, Xiaoxia Zhang

**Affiliations:** 1 Department of Gynecology, First Hospital of Jilin University, No.71 Xinmin Street, Changchun, Jilin Province, 130021, P. R. China; 2 Department of Pathology, First Hospital of Jilin University, No.71 Xinmin Street, Changchun, Jilin Province, 130021, P. R. China; 3 Department of Orthopedics, China-Japan Union Hospital of Jilin University, No.126 Xiantai Street, Changchun, Jilin Province, 130033, P. R. China

**Keywords:** α-fetoprotein, Biomarkers, CA-125, CK7, Hepatoid carcinoma of the ovary, Immunohistochemical staining, p53

## Abstract

Hepatoid carcinoma of the ovary (HCO), a rare invasive malignant tumor composed mainly of epithelioid cells, presented with unilateral or bilateral ovarian masses and elevated serum α-fetoprotein (AFP), has been found mainly in post-menopausal women. We hereby report on the case of a 53-year-old Chinese woman who presented with abdominal distension and a lower abdominal mass with high serum levels of CA-125 and AFP. She was later diagnosed with bilateral HCO. After surgery and following chemotherapy, the patient had no recurrence of tumor or ascites. The hepatoid cells were positive for AFP, p53 and CK7 by immunohistochemistry. Her serum CA-125 and AFP levels had decreased significantly after surgery. Our results suggest that testing and monitoring of serum levels of AFP and CA-125 are considered as potential biomarkers in the diagnosis and progression of this malignancy, and that tissue immunohistochemical staining for AFP, p53 and CK7, plays an important role in distinguishing HCO from other ovarian tumors.

## Background

Hepatoid carcinomas are less frequently occurring tumors of the stomach, lungs, kidneys, endometrium and ovaries, with pathological features similar to those of hepatocellular carcinoma (HCC). Hepatoid carcinoma of the ovary (HCO) is a very rare type of high-grade, invasive malignant ovarian tumor composed mainly of epithelioid cells. This malignancy has been reported mainly in post-menopausal women presenting with unilateral or bilateral ovarian masses and elevated serum level of α-fetoprotein (AFP). Microscopically, these tumors are characterized by solid sheets of large cells with abundant eosinophilic cytoplasm, centrally pleomorphic nuclei and distinct cellular borders. The tumor cells are diffusely immunoreactive for AFP. To date, only 20 patients with diagnosis of HCO have been reported. We describe here the case of a 53-year-old Chinese woman diagnosed with a primary HCO, the clinical and pathological characteristics are also briefly discussed.

## Case presentation

**Figure 1:**
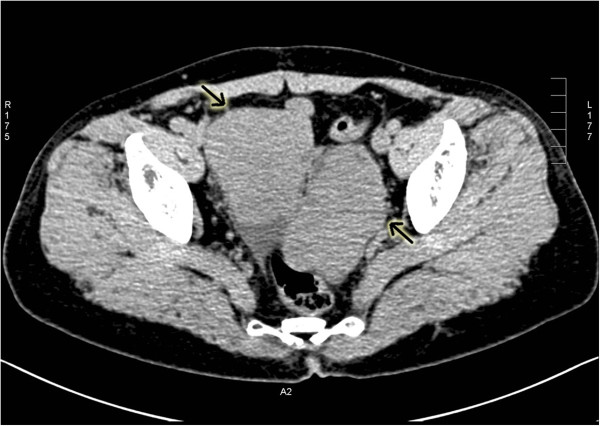
**Abdominal CT scans in the patient.** Abdominal CT scan was performed in the patient and showed two soft tissue masses in the pelvic cavity (one measuring 7.4×6.4 cm and the other measuring 8.0×4.6 cm). No abnormalities were seen in the bladder, liver, cervical stump or rectum. The arrows indicated the messes.

A 53-year-old Chinese woman was admitted to our hospital due to abdominal distension for 1 month and a lower abdominal mass for 10 days. She was diagnosed with hysteromyoma and underwent a sub-total hysterectomy. There was no familial history of gynecological disease. Her father died of liver disease. Pelvic examination revealed two palpable, connected masses, irregular in shape, tender to touch and measuring 8×6×4 cm. Color Doppler ultrasound showed that the uterus was absent and the cervical stump was smooth. Two heterogeneous hypoechoic foci of irregular shape were observed in the uterus, one measuring 9.2×7.1 cm in the right adnexa and the other measuring 8.0×6.7 cm in the left adnexa. A computed tomography (CT) scan of the abdomen showed two soft tissue masses in the pelvic cavity, measuring 7.4×6.4 cm and 8.0×4.6 cm. No abnormalities were seen in the bladder, liver, cervical stump or rectum (Figure 1). Her serum CA-125 level was 124.60 U/mL (normal <35 U/mL) and her serum AFP concentration was 761.20 ng/mL (normal <7.0 ng/mL). Her serum levels of carcinoembryonic antigen (CEA), CA-199, estradiol and testosterone, were within normal ranges.

She was diagnosed with an ovarian malignant tumor and underwent bilateral adnexa dissection, greater omentum dissection and dissection of metastatic nodules on the mesentery. About 40 mL of pale yellow liquid was found in the abdominal cavity. On gross examination, the right ovary was enlarged to 9×7×6 cm and the left ovary to 7×7×6 cm, with both ovaries irregular in shape. Several cauliflower lesions were presented on the surface of both ovaries, with several hard, white nodules, measuring 5 to 20 mm in diameter, on the surface of the greater omentum and mesentery. The cut surface of the tumor was mostly solid and yellow-brown, with several gray-white hard nodules on the surface of the greater omentum. The surface of liver was negative for metastasis.

**Figure 2:**
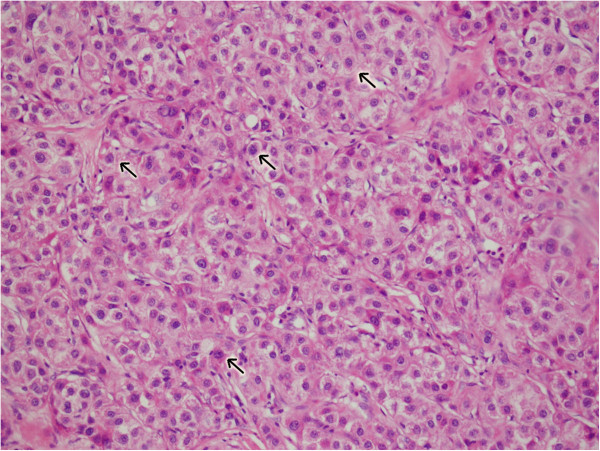
**Light microscopic examination of the tumor cells.** The tumors removed were processed and slides were washed and counterstained with H&E stain (200x), observed under light microscopy showing polygonal and oval in shape. The tumors were composed of sheets, nests and trabeculae of cells with distinct borders and containing moderate to abundant amounts of eosinophilic cytoplasm. The nuclei were oval and located in the center of the cells. The arrows indicate the tumor cells.

**Figure 3:**
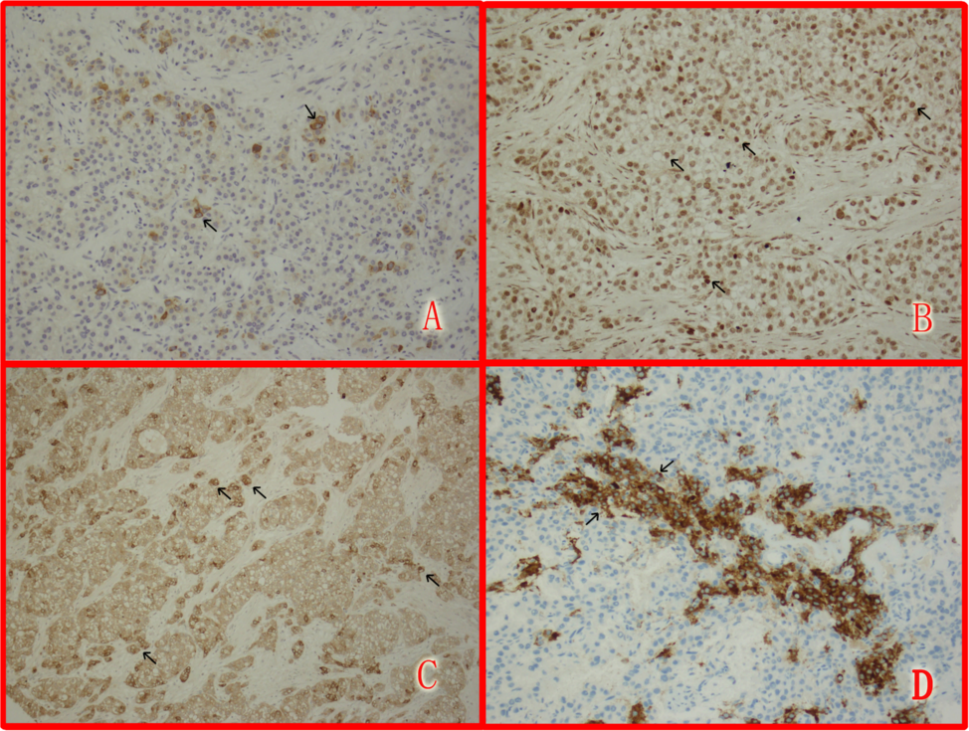
**Immunohistochemical staining of AFP, p53, CK7 and α-inhibin in tumor cells.** Tumor tissues were inflated, fixed in formalin, paraffin-embedded and sectioned for histological analysis. Slides were washed and counterstained with antibodies to AFP, p53, CK7 and α-inhibin using immunohistochemistry staining kits (Maxin BIO, Fu Zhou, China). The results showed that the hepatoid cells were focally positive for AFP (**A**), positive for p53 (**B**) and CK7 (**C**), and α-inhibin (**D**)) (Original magnification, 200x).

Light microscopic examination showed that the tumor cells were polygonal and oval in shape and that the tumors were composed of sheets of cells with moderate to abundant amounts of eosinophilic cytoplasm. The nuclei were oval and located at the center of the cells. Most tumor cells had distinct borders and were arranged in sheets, nests or trabeculae (Figure 2). Paraffin tissue blocks were prepared and stained with hematoxylin-eosin and antibodies to AFP, AAT, CD99, CEA, polyclonal CEA, CA-125, HCG, α-inhibin, CK7, CK8, CK20, EMA, CD10 and WT1. The results showed that hepatoid cells were positive for AFP, p53 and CK7 and focally positive for α-inhibin, but negative for CK20, CDX-2, Vimentin, CA-125, OCT3/4, NSE, hepatocyte paraffin 1, WT1, PLAP, AG/AG, polyclonal CEA, EMA, CD10 and CgA (Figure 3). The percentage of tumor cells that were positive for CK7 was greater than 90%. In contrast, less than 10% of tumor cells were positive for AFP. She was later confirmed with bilateral HCO with involvement of the greater omentum.

Seven days after surgery, she was started on chemotherapy with paclitaxel and carboplatin, administered once every 21 days. Three weeks later, serum levels of CA-125 and AFP decreased to 55.75 U/mL and 120.30 ng/mL, respectively. She received a total of 6 cycles of chemotherapy. Follow-up examination with color Doppler ultrasound of the abdomen and pelvic cavity 15 months after treatment showed no recurrence of tumor and ascites. Her serum CA-125 and AFP levels decreased to 10.87 U/mL and 113.30 ng/mL, respectively.

## Discussion

The first case of HCO was described in 1987. Originally, HCO, a rare ovarian tumor that usually presents with signs and symptoms of an adnexal mass, such as progressive abdominal distension and lower abdominal pain, has been described in only post-menopausal women aged from 42 to 78 years (average, 63 years) [1]. It can also be detected as an asymptomatic unilateral ovarian mass (rarely bilateral). This malignancy mostly was found at an advanced clinical stage and progresses rapidly, with metastases to the abdomen and occasionally to the lungs; with median survival about 2 years [2].

Macroscopically, HCOs are solid and cystic with a size of 20 cm in the greatest diameter. Histologically, HCO resembles HCC, which are composed of solid sheets or aggregates of uniform cells with moderate and abundant acidophilic cytoplasm, distinct cell borders and centrally located, prominent nuclei. Mitoses, some even atypical, are frequent [3]. HCO is associated with elevated serum AFP and CA-125 levels. At diagnosis, our patient had serum CA-125 concentration of 124.60 U/mL and AFP concentration of 761.20 ng/mL. After the first cycle of chemotherapy, the CA-125 and AFP levels decreased to 55.75 U/mL and 120.30 ng/mL, respectively. After 15 months and 6 cycles of chemotherapy, they both reduced to 10.87 U/mL and 113.30 ng/mL, this suggested that follow-up by monitoring serum AFP and CA-125 levels was an important prognostic indicator in this patient. Immunohistochemical staining showed this tumor to be positive for AFP, CEA and cytokeratin. Although the tumor was negative for EMA and CD10, which may show a canalicular pattern, this type of staining is not essential for diagnosis of hepatoid carcinoma and our clinical, morphological and immunohistochemical findings collectively support the diagnosis.

Normal hepatocytes, bile duct epithelium and HCCs are focally positive for CK18, as shown in HCOs. In contrast, CK19 and CK20 are expressed in normal gastrointestinal epithelial cells, bile duct cells and some adenocarcinomas including ovarian surface epithelial carcinomas, but not in normal hepatocytes and HCCs [4]. The CK profile of HCO differed from that of normal and neoplastic hepatocytes, but was similar to that of common epithelial adenocarcinomas, which were positive for CK19 and CK 20, suggesting that HCOs might come from epithelial origin [5]. Study of the expression of hepatocyte paraffin 1, which reacted with normal and neoplastic hepatocytes in ovarian tumors with hepatoid differentiation, showed that expression of this protein was correlated with the degree of hepatoid differentiation [4]. Thus, diagnosis of HCO was based on histopathological findings, immunohistochemical staining patterns and marked elevation of AFP.

Differential diagnosis of HCO includes yolk sac tumors of the ovary and endometrial carcinomas. Yolk sac tumors of the ovary are rare but highly likely to be malignant, and are often bulky and increase rapidly in size. They express high amounts of AFP, which increases with tumor progression. Yolk sac tumors of the ovary are difficult to diagnose. Thus, multiple examinations of pathological slides are needed to ensure that these are not mixed tumors, especially tumors containing teratomas [6]. In the clinical setting, the presence of these globules, along with the production of AFP, can be diagnosed as yolk sac tumors of the ovary. Endometrial adenocarcinomas rarely produce AFP. Microscopically, endometrial adenocarcinomas are usually composed of a major medullary portion and a minor tubular adenocarcinoma invading the myometrium, myometrial lymphatics and blood vessels. Vascular permeation by neoplastic cells was prominent and extensive hepatoma-like features were often observed [7].

To date, there is insufficient data regarding the optimal treatment of patients with HCO. Most patients are treated by surgery, followed by a chemotherapy regimen similar to those used in patients with ovarian-like carcinomas. HCOs are highly aggressive, with 75% of patients presenting with stage III or IV diseases. For these patients, the initial step in patient management is reduction of the surgical bulk, followed by chemotherapy. In this case, the current treatment of choice for initial chemotherapy is six cycles of paclitaxel plus carboplatin. Additional efficacy may be provided by intraperitoneal chemotherapy through the combination of new cytotoxic agents with paclitaxel and carboplatin, and integrating biological agents into front-line therapy [8]. Even after surgery and ovarian-like cancer chemotherapy regimens, such as carboplatin and paclitaxel, most patients showed limited responses. Patients with immunological disorders tend to have a poor tolerance to chemotherapy and short survival, as reported in a 46-year-old African-American woman with HCO and systemic lupus erythematosus [4]. Our patient was treated with surgery followed by a total of six cycles of paclitaxel and carboplatin chemotherapy. We found that this regimen was well tolerated and toxicities were manageable. Follow-up color Doppler ultrasound examination of the abdomen and pelvic cavity in this patient showed no evidence of tumor recurrence or ascites 15 months after initial diagnosis.

## Conclusions

In summary, we report a rare case of HCO in a 53-year-old Chinese woman. Our results show that surgery followed by administration of a total of six cycles of paclitaxel and carboplatin chemotherapy, was a reasonable approach for this patient. Examination of the serum AFP and CA-125 levels is important for the diagnosis and prognosis of this disease. In addition, immunohistochemical staining for AFP, p53 and CK7 are helpful in distinguishing HCOs from other ovarian tumors with hepatoid features.

## Consent

Written informed consent and any accompanying images were obtained from the patient for publication of this case report.

## Abbreviations

AFP: α-fetoprotein; HCC: Hepatocellular carcinoma; HCO: Hepatoid carcinoma of the ovary; CEA: Carcinoembryonic antigen; CT: Computed tomography.

## Competing interests

The authors declare that they have no competing interests.

## Authors’ contributions

LW performed the case analysis and drafted the manuscript; YZ carried out the pathological analysis and immunohistological experiments; LS was involved in the acquisition of data and the patient follow-up; HZ participated in the immunohistological experiments; WC conceived the study design and helped to draft the manuscript; XZ drafted and revised the manuscript. All authors read and approved the final manuscript.
